# A High Level of Soluble CD40L Is Associated with *P*. *aeruginosa* Infection in Patients with Cystic Fibrosis

**DOI:** 10.1371/journal.pone.0168819

**Published:** 2016-12-28

**Authors:** Adriana Ester Bustamante, José Carlos Jaime-Pérez, Paula Cordero-Pérez, Gabriela Galindo-Rodríguez, Linda Elsa Muñoz-Espinosa, César Daniel Villarreal-Villarreal, Roberto Mercado-Longoria

**Affiliations:** 1 Clínica de Fibrosis Quística, Servicio de Neumología, Universidad Autónoma de Nuevo León, Facultad de Medicina, Hospital Universitario “Dr. José E. González”, Monterrey Nuevo León, México; 2 Departamento de Hematología, Universidad Autónoma de Nuevo León, Facultad de Medicina, Hospital Universitario “Dr. José E. González”, Monterrey Nuevo León, México; 3 Unidad de Hígado, Universidad Autónoma de Nuevo León, Facultad de Medicina, Hospital Universitario “Dr. José E. González”, Monterrey Nuevo León, México; 4 Servicio de Alergia e Inmunología, Universidad Autónoma de Nuevo León, Facultad de Medicina, Hospital Universitario “Dr. José E. González”, Monterrey Nuevo León, México; Centre National de la Recherche Scientifique, FRANCE

## Abstract

**Objective:**

The aim of this study was to evaluate whether the concentration of sCD40L, a product of platelet activation, correlates with the presence of *Pseudomonas aeruginosa* in the airway of patients with cystic fibrosis (CF) and to determine its possible clinical association.

**Methods:**

Sixty patients with CF, ranging in age from 2 months to 36 years, were studied. The demographics, cystic fibrosis transmembrane conductance regulator (CFTR) genotype, spirometry measurements, radiographic and tomographic scans, platelet count in peripheral blood, sCD40L, IL-6, TNF-α and ICAM1 data were collected. Infection-colonization of the airway was evaluated using sputum and throat swab cultures; the levels of anti-*Pseudomonas aeruginosa* antibodies (Anti-PaAb) were evaluated.

**Results:**

Patients with CF and chronic colonization had anti-PaAb values of 11.6 ± 2.1 ELISA units (EU) and sCD40L in plasma of 1530.9 ±1162.3 pg/mL; those with intermittent infection had 5.7 ± 2.7 EU and 2243.6 ± 1475.9 pg/mL; and those who were never infected had 3.46 ± 1.8 EU (p≤0.001) and 1008.1 ± 746.8 pg/mL (p≤0.01), respectively. The cutoff value of sCD40L of 1255 pg/mL was associated with an area under the ROC (receiver operating characteristic curve) of 0.84 (95% CI, 0.71 to 0.97), reflecting *P*. *aeruginosa* infection with a sensitivity of 73% and a specificity of 89%. Lung damage was determined using the Brasfield Score, the Bhalla Score, and spirometry (FVC%, FEV1%) and found to be significantly different among the groups (p≤0.001).

**Conclusion:**

Circulating sCD40L levels are increased in patients with cystic fibrosis and *P*. *aeruginosa* infection. Soluble CD40L appears to reflect infection and provides a tool for monitoring the evolution of lung deterioration.

## Introduction

Cystic fibrosis (CF) is one of the most common inherited diseases resulting in a shortened life span. It affects approximately 70,000 people worldwide [1–2]. CF is an autosomal recessive disease that is caused by mutations in the gene encoding the CF transmembrane conductance regulator (CFTR), located on the long arm of the chromosome 7[3]. There are more than 2000 CFTR mutations. At the airway level, these mutations lead to defects in the CFTR causing reduced chloride secretion, which favors the reabsorption of sodium and results in dried secretions, poor mucociliary clearance, and airway obstruction [3–4].

Lung disease in CF, characterized by chronic endobronchial infection with specific pathogens, is associated with inflammation dominated by neutrophils that contribute to progressive lung damage [5–7]. Numerous studies have shown that both infection and inflammation begin early in life [8–9], and they represent a determining factor for the deteriorating lung function and shortened survival. *P*. *aeruginosa* is the main pathogen that infects patients with CF [10–11].

Controversy exists regarding whether lung inflammation is present in the CF lung even without infection or whether inflammation is an exaggerated response to sustained bacterial infections. Undoubtedly, lung inflammation is more intense in the presence of chronic infection [5–6, 8]. Regardless of whether inflammation precedes or follows infection in patients with CF, the inflammation is severe. Research has indicated that platelets contribute significantly to the incidence of inflammation [12].

Platelets secrete co-stimulatory molecules such as CD40L. The soluble form of CD40L (sCD40L) in human circulation is nearly entirely derived from platelets [13]. This study evaluated the presence of anti-*P*. *aeruginosa* antibodies (anti-PaAb) and the concentration of sCD40L and their relationship with *P*. *aeruginosa* infection in a cohort of patients with cystic fibrosis.

## Materials and Methods

### Study design and population

The participants were recruited from the CF clinic of the “Dr. José Eleuterio González” School of Medicine and University Hospital of the Autonomous University of Nuevo Leon in Monterrey, Mexico, from November 2011 to May 2013. The Ethics and Research Committees of the University Hospital José Eleuterio González (Registration no. NM11015) approved this study protocol. Written informed consent was obtained from either the subjects or their caregivers.

We included all patients with a positive diagnosis of CF using electrolytes in sweat, which was performed with pilocarpine iontophoresis (chloride in sweat ≥ 60 mmol/L) and adhered to the procedures from a genetic study using two mutations in CFTR known to cause cystic fibrosis; patients with advanced liver disease or use of systemic corticosteroids were excluded.

This ambispective observational study aimed to assess patients with cystic fibrosis and implement interventions according to standard international guidelines. For our protocol, the patients were divided according to Leeds criteria into the following groups [14]: 1) patients with chronic *P*. *aeruginosa* colonization; 2) patients with intermittent infection by the same bacteria; and 3) patients in whom *P*. *aeruginosa* was never identified.

### Review of clinical records

We created a database that included demographic parameters; age; sex; age at the time of diagnosis; clinical, biochemical, functional and imaging test results; identified mutations; a history of colonization with *P*. *aeruginosa* (during the previous year); cultures of sputum and throat swabs; radiographic score; tomographic score; and pulmonary function tests.

### Follow-up of patients with CF

The follow-up protocol in the CF clinic included monitoring visits at least four times throughout the year, during which nutritional status was assessed by weight and height: weight/height ratio in children under 2 years and/or body mass index (IMC) in individuals over 2 years; also, respiratory clinical symptoms and oxygen saturation by pulse oximetry were assessed, and once a year, blood chemistry and chest X-ray were performed. The chest radiograph was evaluated using the classic radiographic CF score, the Brasfield Radiological Scoring System [15]. From 3 years of age and afterwards, every three years, high-resolution chest tomography was performed and evaluated using the Bhalla score [16].

A respiratory sample was obtained at the initial visit and repeated every three months. Additional samples were obtained if the patient had respiratory symptoms or at the end of eradication treatment. The samples were obtained by spontaneous expectoration orby deep throat swab.

Sputum samples were plated on chocolate agar, EMB agar, sodium azide agar, staphylococcus S110 agar and blood agar. Throat swabs were plated on sheep blood agar. The blood agar and chocolate agar plates were incubated in micro-aerobiosis, and the remainder were incubated aerobically for up to 72 h at 36°C. Bacterial colonies were identified using conventional biochemical tests and the biochemical system API20 NE (BioMerieux, Inc., Durham, NC, USA).

Pulmonary function tests were performed by spirometry following the guidelines of the American Thoracic Society [17]. The tests were conducted using the Platinum EliteSeries^™^, BreezeSuite^™^ version 7.2 SP3 plethysmograph (MGC Diagnostics, St. Paul, MN, USA). The results were expressed as the percentage of the predicted values for FEV1 and FVC by sex, age and height.

The patients were treated with the usual indications, including a hypercaloric diet, pancreatic enzymes, fat-soluble vitamins, nebulized bronchodilator, and Pulmozyme NR, secretion clearance therapy, systemic antibiotics, if needed. Treatment was aimed at eradicating *P*. *aeruginosa* in the case of infection, initial or recent, with tobramycin 300 mg twice daily inhaled for 28 days.

For the purposes of this protocol, if the patient was stable, a 7.0-mL sample of peripheral blood was obtained, centrifuged and separated into three aliquots of 1.0 mL of serum and frozen at -70°C to determine the concentration of cytokines (IL-6, ICAM-1, TNF-α, VEGF), sCD40L, and anti-PaAb. A second sample for sCD40L was obtained at the end of the inhaled antibiotic cycle in non-chronic patients in whom positive cultures for *P*. *aeruginosa* were documented within six months of obtaining the baseline sample and in those who received eradication treatment with inhaled tobramycin.

### Analysis of serum parameters

In all patients, a platelet count, the sCD40L fraction, and inflammatory mediators were determined; IL6, ICAM-1, TNF-α, and VEGF were measured using ELISA according to the manufacturer's instructions (Pre-ProTech, México; ASYS Hitech GmbH Expert ELISA Plus UV G020151, Eugendorf, Austria); and specific antibodies against *Pseudomonas aeruginosa* were measured using the Pseudomonas-CF-IgG ELISA kit (Statens Serum Institut SSI Diagnostica, Hillerød, Denmark).

### Statistical analysis

Parametric or non-parametric tests were conducted as appropriate (ANOVA and a homogeneity of numerical variances test). For categorical variables, contingency tables and the chi-squared test were used.

For sCD40L, a ROC (receiver operating curve) analysis, including the area under the curve (AUC), was performed to assess cutoff values for this marker to reflect *P*. *aeruginosa* infection. For sCD40L measurements before and after eradication therapy, a paired *t* test for related samples was performed. All values are reported as the means ± SD with 95% confidence intervals. A *P*<0.05 was considered to be statistically significant. The data were collected in a database and were analyzed using SPSS 20.0 for Windows.

## Results

During the study period, 64 patients were invited to participate, and 60 met the inclusion criteria and signed the informed consent/assent form. Thirty-one women and 29 men with a mean age of 9.5 ± 7.2 years, who regularly attended the CF clinic, were included in the study. The genetic study was conducted in 49/60 (81.7%) patients and at least one mutation in the CFTR was identified, being homozygous for the F508del in 16/60 (26.7%), heterozygous for the F508del mutation in 21/60 (35%), and two different F508del mutations in 12/60 (20%); in the remaining 11 (18.3%) patients, a molecular study was not performed.

According to the Leeds classification [14], 24 (37.5%) patients were chronically colonized with *P*. *aeruginosa*, whereas 26 (40.6%) patients had intermittent infection and had received eradication treatment in each new positive culture for the bacteria according to international guidelines; in 10 (15.6%) patients, *P*. *aeruginosa* was never isolated from the respiratory secretions and throat swab cultures.

Other bacteria detected at least once in the year before the study were methicillin-sensitive *Staphylococcus aureus* in 55 (91%) patients, methicillin-resistant *S*. *aureus* in 3 (5%) patients, *Stenotrophomonas maltophilia* in 3 (5%) patients, *Achromobacter xylosoxidans* in 4 (6%) patients, Enterobacteriaceae in 3 (5%) patients, *Burkholderia Gladiolii* in 1/60 patients and *Aspergillus fumigatus* 2/60 patients. The general characteristics of the group of CF patients are shown in Table 1.

**Table 1:** General characteristics of patients with cystic fibrosis and their bacteriological status in relation to *P*. *aeruginosa* infection.

Characteristics (n = 60)	Chronic *P*. *aeruginosa*	Intermittent *P*. *aeruginosa*	*P*. *aeruginosa* never detected	P
**Age, months (range)**	58.8 (1–292)	22.31 (1–348)	62.1 (1–180)	0.086
**BMI, kg/m**^**2**^	16.5 ± 3.72	15.78 ± 2.46	17 ± 2.23	0.620
**Sex (%)**				
Men	11 (37.9)	11 (37.9)	7 (24.1)	
Women	13 (41.9)	15 (48.4)	3 (9.7)	

Of the 26 patients with intermittent infection and of the 10 uninfected, 17 (47.2%) patients experienced a new or recent infection with *P*. *aeruginosa (*within 6 months of obtaining the baseline sample) and received eradication treatment; a second blood sample was obtained in 13 patients to evaluate the performance of sCD40L at the end of eradication treatment. The difference in mean values of sCD40L, before (2893 pg/mL) and after (2073 pg/mL) eradication treatment was statistically significant (*p<* 0.045).

The relationship between cytokines and markers used in each of the groups, and the levels of anti *P*. *aeruginosa* antibodies and sCD40L had a statistically significant relationship with *P*. *aeruginosa* infection and chronic colonization as shown in Table 2.

**Table 2:** Comparison of cytokines and biomarkers with chronic/non-chronic/never infected *P*. *aeruginosa* in patients with cystic fibrosis.

Cytokines and biomarkers	C*Pa*, n = 24	NC*Pa*, n = 26	NI*Pa*, n = 10	95% CI	P
TNF-α, pg/mL	49.4 ± 15.3	48.38 ± 9.8	59.8 ± 28.8	C*Pa* 42.9–55.9	0.491
				NC*Pa* 44.4–52.4	
				NI*Pa* 39.2–80.4	
IL-6, pg/mL	232.1 ± 388.8	279.3 ± 436.9	301.7 ± 436.9	C*Pa* 67.9–396.3	0.872
				NC*Pa* 116.3–442.3	
				NI*Pa* 10.78–614.3	
ICAM-1, pg/mL	1237 ± 82.7	1229 ± 196.2	1387.1 ± 313.2	C*Pa* 1202–1271.4	0.344
				NC*Pa* 1148–1308.3	
				NI*Pa* 1208.7–1308.6	
VEGF, pg/mL	236.5 ± 382.2	130.8 ± 148.1	88.6 ± 71.3	C*Pa* 75.12–397.8	0.277
				NC*Pa* 71.01–190.62	
				NI*Pa* 37.6–139.7	
sCD40L, pg/mL	1530.9 ± 1162.3	2243.6 ± 1475.9	1008.1 ± 746.8	C*Pa* 1040–2021.7	0.010
				NC*Pa* 1647.4–2839.7	
				NI*Pa* 473.9–1542.5	
Anti-PaAb (EU)	11.60 ± 2.1	5.71 ± 2.68	3.5 ± 1.8	C*Pa* 10.7–12.53	≤ 0.001
				NC*Pa* 4.6–6.8	
				NI*Pa* 2.15–4.77	
Platelets	363.9 ± 120.2	369.73 ± 95.31	306.1 ± 79.1	C*Pa* 313.2–414.7	0.243
				NC*Pa* 331.2–408.3	
				NI*Pa* 329–383.84	

TNF-α, tumor necrosis factor alpha; IL-6 Interleukin-6; ICAM-1, intracellular adhesion molecule-1; VEGF, vascular endothelial growth factor; sCD40L, soluble CD40 ligand; anti-PaAb, anti-*P*. *aeruginosa* antibody; C*Pa*, chronic *P*. *aeruginosa*; NC*Pa*, non-chronic *P*. *aeruginosa*; NI*Pa*, never infected *P*. *aeruginosa*.

Control values for sCD40L: 160–1000 pg/mL.

The impact of *P*. *aeruginosa* infection-colonization on pulmonary deterioration was measured using pulmonary function tests and radiographic and tomographic studies and is shown in Table 3.

**Table 3:** Comparison of chronic/non-chronic/never infected *P*. *aeruginosa* infection vs. lung function and imaging studies in patients with cystic fibrosis.

Lung function	C*Pa* (n = 23)	NC*Pa* (n = 17)	NI*Pa* (n = 6)	95% CI	P
**FEV1%, n = 46/60**	57 ± 26	88 ± 16	85 ± 19	C*Pa* 45–68	≤0.001
				NC*Pa* 79–96	
				NI*Pa* 64–106	
**FVC%, n = 46/60**	69 ± 21	90 ± 15	94 ± 9	C*Pa* 59–79	≤0.001
				NC*Pa* 82–98	
				NI*Pa* 84–104	
Bronchiectasis in CT (Balha)*	4.2 ± 1.9	1.72 ± 1.8	1.4 ± 1.8	C*Pa* 3.35–5.1	≤0.001
				NC*Pa* .81–2.64	
				NI*Pa* 1.7–2.92	
Balha CT score*	11.2 ± 5.1	5.5 ± 4.7	3.9 ± 4.1	C*Pa* 8.9–13.5	≤0.001
				NC*Pa* 3.18–7.8	
				NI*Pa* 0.46–7.3	
**Brasfield, chest X-ray, n = 60**	17.9 ± 3.4	22.8 ± 2.1	23.4 ± 2.2	C*Pa* 16.5–19.4	≤0.001
				NC*Pa* 21.4–23.1	
				NI*Pa* 21.9–24.9	

*n = 48/60 FEV1, forced expiratory volume in 1 second; FVC, forced vital capacity, C*Pa* chronic *P*. *aeruginosa*, NC*Pa*, non-chronic *P*. *aeruginosa*; NI*Pa*, never infected *P*. *aeruginosa*.

**Fig 1:**
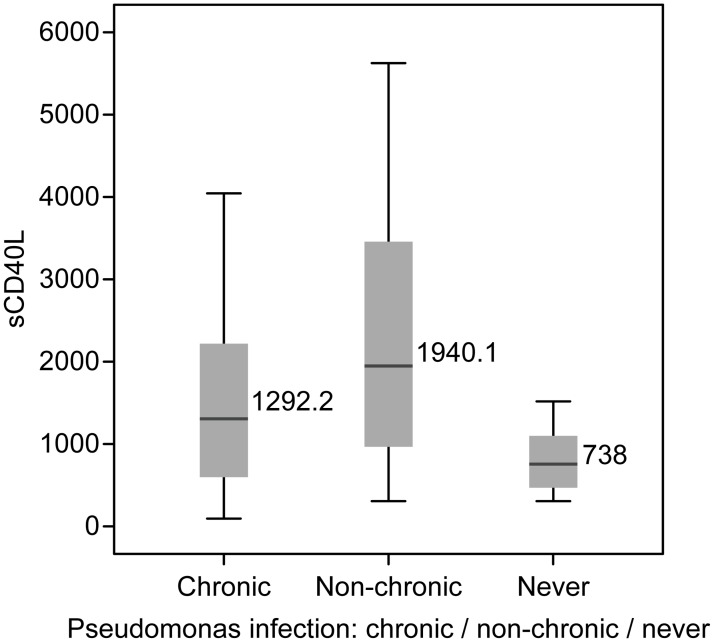
Medians of sCD40L of the three groups of patients with cystic fibrosis.

The medians of sCD40L for each of the evaluated groups are shown in Fig 1.

**Fig 2:**
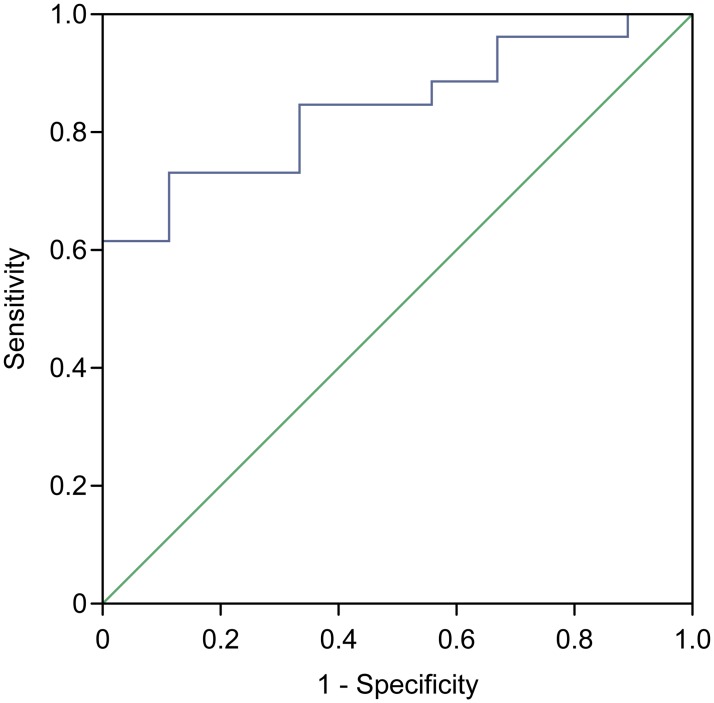
ROC curve showing the characteristics of the association between the sCD40L levels and the presence of *P*. *aeruginosa* in patients with cystic fibrosis. Area under the curve (AUC) was 0.84 (95% CI, 0.71–0.97).

The sCD40L performance was optimized using a cutoff of 1255 pg/mL, with an area under the ROC curve of 0.84 (95% CI, 0.71–0.89) (Fig 2).

There was no individual correlation between sCD40L and platelet counts in any of the groups; in contrast, there was a statistically significant moderate negative correlation between AcPa and FEV1 (Pearson = .553; p = 0.001), and between AcPa and FVC (Pearson = .537; p = 0.001).

## Discussion

Lung disease in CF is characterized by progressive destruction of lung tissue due to bacterial infection, inflammation, and airway remodeling [18–19]. Multiple alterations in both innate and acquired immunity in patients with CF have been demonstrated [20–33]. Pathogen-host interactions are important for explaining the severity and persistence of the inflammatory process, with *P*. *aeruginosa* being the predominant pathogen.

The difficulty in obtaining a representative sample of the lower respiratory airway in infants and young children demonstrates the need for methods that can complement or be an alternative to cultures [34]. An example of ancillary methods is the detection of serum antibodies against *P*. *aeruginosa*; however, its use in clinical practice remains controversial [35]. In this study, anti-PaAb showed a positive correlation with sCD40L levels, suggesting that its fluctuation reliably reflects the clinical status of infection by this bacterium. In this case, the search for biomarkers of lung damage and the presence of *P*. *aeruginosa* in the airway of patients with CF is of utmost importance for follow-up and therapeutic management.

In CF patients, platelet activation has been shown to occur and correlates with lung involvement [12, 36]. Ninety-five percent of sCD40L fraction is released by activated platelets [13] and its interaction with the CD40 receptor plays an important role in the induction of efficient humoral and cellular immune responses. The co-stimulatory molecules, including CD40L, are capable of regulating inflammation, and their blood levels as well as their soluble isoforms may serve as markers of activity in diverse pathological conditions, including sepsis [37], tuberculosis [38], and parasitic diseases [39], among others. Patients with CF have an intense systemic inflammation in which platelets are involved. Platelets can be activated by antigen-antibody complexes, microorganisms and endotoxins, including the lipopolysaccharide of *P*. *aeruginosa* [40–41]. After activation, they interact with endothelial cells and circulating leukocytes through mediators, contributing significantly to the initiation and spread of diseases and inflammatory processes such as CF lung disease. Given the observed increase in the platelet count of our patients with CF and *P*. *aeruginosa* infection, we sought to demonstrate the activation of these immune cells in this context by determining sCD40L levels in the blood, finding a correlation between these biomarkers and their concentration and the clinical status of patients with CF.

Normal sCD40L values in healthy controls range from 0.16 ng/mL to 10 ng/mL[42]. Falco et al. [36] showed that patients with CF have increased sCD40L levels in circulating blood compared to healthy controls and that these levels are inversely related to the deterioration of lung function assessed through FEV1, although in this study, all patients, except one, had chronic colonization with *P*. *aeruginosa*.

The patients in our study were divided into three groups according to the status of infection by *P*. *aeruginosa*, allowing us to assess the relationship of sCD40L with the presence or absence of the pathogen. The patients with CF showed changes in expression of soluble CD40L; these changes were associated with the presence of *P*. *aeruginosa* in cultures. sCD40L efficiency was optimized using a cutoff of 1255 pg/mL with an area under the ROC curve of 0.84 (95% CI, 0.71–0.89) for the identification of patients with *P*. *aeruginosa* infection. In those without chronic *P*. *aeruginosa* colonization and with a positive culture within six months of the initial sample, eradication treatment was conducted with inhaled tobramycin, and a second sample was obtained at the end of treatment, documenting a statistically significant reduction (*p<*0.045) in sCD40L levels, suggesting that the severity of infection by *P*. *aeruginosa* in CF is dynamically reflected by the concentration of sCD40L.

Furthermore, the negative correlation between anti-PaAb and lung function (FEV1 and FVC) shows lung damage as a result of *P*. *aeruginosa* chronic colonization.

The sCD40L levels were higher in the group of patients with intermittent infection than in those with chronic colonization. First, one likely explanation is that most chronically colonized patients were treated with inhaled tobramycin in ON-OFF cycles, which can reduce inflammation [43] and sCD40L levels. This explanation suggests that serially measuring the serum level of sCD40L in CF patients may be useful in the follow-up of patients and may be helpful in making treatment decisions; the persistence of high levels of sCD40L could reflect a lack of response or resistance to treatment and the need to change or intensify it.

In conclusion, these findings suggest that there is an association between sCD40L levels and *P*. *aeruginosa* infection in patients with CF. Soluble CD40L appears to reflect infection status with this bacterium, in addition to providing an additional tool for monitoring the evolution of lung deterioration.
